# Dose optimization of sacubitril/valsartan in Chinese chronic heart failure patients: integration of physiologically based pharmacokinetic modeling and real-world data

**DOI:** 10.3389/fphar.2026.1875094

**Published:** 2026-07-22

**Authors:** Ke Ren, Ming Fan, Mengqi Jia, Zi Wang, Xiao Zhu, Bing Han, Xiaoqiang Xiang, Yanxia Zhang, Qingfeng He

**Affiliations:** 1 Minhang Hospital and School of Pharmacy, Fudan University, Shanghai, China; 2 Department of Pharmacy, Zhongshan Hospital, Fudan University, Shanghai, China; 3 State Key Laboratory of Advanced Drug Formulations for Overcoming Delivery Barriers, Fudan University, Shanghai, China; 4 Hunan Key Laboratory for Bioanalysis of Complex Matrix Samples, Changsha, Hunan, China

**Keywords:** chronic heart failure, dose optimization, PBPK model, real-world data, sacubitril/valsartan

## Abstract

**Objective:**

In routine clinical practice, many patients with chronic heart failure (CHF) treated with sacubitril/valsartan do not achieve the guideline-recommended target dose of 200 mg twice daily (BID), largely because of tolerability concerns. This study aimed to develop a model-informed framework to evaluate whether sacubitril/valsartan 100 mg BID may provide adequate expected pharmacodynamic benefit with improved tolerability in Chinese CHF patients who are unable to tolerate further dose escalation.

**Methods:**

A physiologically based pharmacokinetic (PBPK) model of sacubitril/valsartan was developed in PK-Sim and evaluated against observed clinical data. Physiological changes were incorporated to extrapolate the model to healthy Chinese subjects, patients with renal impairment, and patients with CHF. Real-world clinical data from Chinese CHF patients with hypertension were used to inform virtual population. PBPK-predicted steady-state exposure metrics were then linked to externally sourced exposure–biomarker relationships for NT-proBNP and to a blood pressure response model to compare expected biomarker response and hypotension-related tolerability across dosing strategies.

**Results:**

The PBPK model adequately described the pharmacokinetic profiles of sacubitril, sacubitrilat, and valsartan across different populations and dosing regimens. Overall, 88.75% of predicted-to-observed exposure ratios were within 1.5-fold and 98.75% were within the conventional two-fold boundary, with average fold error and absolute average fold error values below 2. CHF and renal impairment were associated with increased systemic exposure, particularly for sacubitrilat, consistent with its renal elimination pathway. In under-titrated CHF patients, exposure–biomarker translation indicated limited additional NT-proBNP reduction when the regimen was escalated from 100 mg BID to 200 mg BID, suggesting a relatively flat exposure–biomarker relationship over this dose range. In contrast, escalation to 200 mg BID was associated with greater predicted systolic blood pressure reduction and greater hypotension-related tolerability concern.

**Conclusion:**

This PBPK-based model-informed framework supports the guideline-recommended target dose of sacubitril/valsartan 200 mg BID when tolerated, while providing exploratory model-informed evidence that 100 mg BID may represent a feasible alternative for patients with limited tolerance to further dose escalation. Prospective validation using longitudinal biomarker, tolerability, and clinical outcome data is warranted.

## Introduction

Chronic heart failure (CHF) is a progressive clinical syndrome caused by structural or functional cardiac abnormalities that impair ventricular filling or ejection and ultimately lead to inadequate systemic perfusion (Cannata et al., 2026). In China, the burden of heart failure has continued to increase over recent decades, driven by population aging, improved survival from cardiovascular diseases, and the rising prevalence of cardiometabolic risk factors (Yang et al., 2025). Recent national analyses further suggest that heart failure is no longer limited to older adults, with an increasing disease burden observed in younger populations (Wang et al., 2025). Despite advances in guideline-directed medical therapy, substantial regional differences and implementation gaps remain in routine heart failure management in China, particularly in the initiation, titration, and long-term maintenance of evidence-based therapies (Zhang et al., 2023).

Sacubitril/valsartan, the first angiotensin receptor–neprilysin inhibitor, has become one of the recommended therapies, particularly for heart failure with reduced ejection fraction (Nasrallah et al., 2024). By combining neprilysin inhibition with angiotensin II receptor blockade, sacubitril/valsartan enhances natriuretic peptide signaling while suppressing the renin–angiotensin–aldosterone system (RAAS) (Zile et al., 2016). Beyond conventional RAAS inhibition, angiotensin receptor–neprilysin inhibitor (ARNI) therapy improves neurohormonal balance, enhances cGMP-mediated cardiovascular effects, and has been associated with favorable changes in cardiac biomarkers, hemodynamic function, and clinical outcomes across diverse patient populations (Inoue et al., 2026; Jin and Yang, 2026; Maur et al., 2026). In the PARADIGM-HF trial, sacubitril/valsartan reduced the risk of cardiovascular death and heart failure hospitalization compared with enalapril, supporting its role as a disease-modifying treatment in chronic heart failure (McMurray John et al., 2014). Current heart failure guidelines generally recommend stepwise titration toward the target dose of 200 mg twice daily when tolerated (Chinese Soci ety of Cardiology et al., 2024). However, in routine clinical practice, many patients do not reach the target dose, and lower maintenance doses are frequently used because of symptomatic hypotension, renal dysfunction, advanced age, or other tolerability concerns (Chen et al., 2021; Proudfoot et al., 2021). Recent clinical evidence further suggests that low baseline systolic blood pressure and more advanced heart failure status are associated with an increased risk of treatment-related hypotension, which may contribute to difficulties in achieving recommended target doses (Gupta et al., 2026; Ando et al., 2026). This real-world under-titration is particularly relevant in Chinese patients, among whom body size, comorbidities, blood pressure reserve, and renal function may differ from trial populations (Guan et al., 2023).

From a clinical pharmacology perspective, dose optimization of sacubitril/valsartan requires simultaneous consideration of efficacy and tolerability. N-terminal pro-BNP (NT-proBNP) is a clinically relevant biomarker of heart failure status and prognosis, and reductions in NT-proBNP during sacubitril/valsartan treatment have been associated with improved clinical outcomes (Zile et al., 2016; Pieske et al., 2021). In contrast, systolic blood pressure is a key safety-related marker during dose escalation, because excessive blood pressure reduction may limit treatment persistence and prevent patients from achieving or maintaining higher doses. Therefore, integrating exposure, NT-proBNP response, and blood pressure effects may provide an exploratory quantitative framework to evaluate whether lower-dose regimens can preserve expected pharmacodynamic benefit while reducing hypotension-related risk.

The pharmacokinetics of sacubitril/valsartan are mechanistically complex and may be influenced by disease-related physiological alterations. After oral administration, the co-crystal formulation dissociates into sacubitril and valsartan. Sacubitril is rapidly hydrolyzed by carboxylesterase 1 to the active neprilysin inhibitor sacubitrilat, which is mainly eliminated through renal pathways, whereas valsartan is primarily cleared through hepatobiliary excretion (Flarakos et al., 2016; Waldmeier et al., 1997). In CHF patients, reduced cardiac output, altered hepatic and renal blood flow, renal impairment, and comorbid hypertension may alter systemic exposure to sacubitrilat and valsartan (Butranova et al., 2025). These changes are difficult to evaluate fully using conventional clinical trials alone, especially in special populations such as elderly patients and those with renal dysfunction.

Physiologically based pharmacokinetic modeling (PBPK) provides a mechanistic approach to integrate drug-specific properties with population- and disease-specific physiological parameters, including organ size, blood flow, renal function, transport processes, and demographic characteristics (Grimstein et al., 2019). PBPK models have been increasingly used in regulatory submissions to support dose selection, drug–drug interaction assessment, and extrapolation to special populations. The FDA and EMA have both issued guidance on the reporting and qualification of PBPK analyses, and the ICH M15 guideline further emphasizes the role of model-informed drug development evidence in supporting dose optimization and benefit–risk assessment (U.S. Food and Drug Administration and Center for Drug Evaluation and Research, 2018; Li et al., 2025; Sun et al., 2024; Rodrigues et al., 2025; Marshall et al., 2023). In this context, PBPK modeling is well suited to investigate sacubitril/valsartan exposure in Chinese CHF patients and clinically relevant subgroups where direct pharmacokinetic evidence is limited.

In this study, we developed and evaluated a PBPK model of sacubitril/valsartan and applied it to Chinese patients with CHF and relevant comorbid subpopulations, including hypertension and renal impairment. Model-predicted exposure metrics were further linked to externally sourced exposure–biomarker relationships for NT-proBNP and to blood pressure response models to compare alternative dosing strategies. The objective was not to replace guideline-recommended target dosing, but to generate model-informed evidence on whether sacubitril/valsartan 100 mg twice daily may represent a clinically feasible regimen for Chinese CHF patients with limited tolerance to further dose escalation.

## Materials and methods

### Modeling software and overall workflow

The PBPK models were developed using the PK-Sim and MoBi (version 12.1, 2025, www.open‐systems‐pharmacology.org) modeling software, which are components of the Open Systems Pharmacology (OSP) Suite. Parameter identification and sensitivity analysis were performed with PK-Sim. Published concentration–time curves were digitized using WebPlotDigitizer (https://apps.automeris.io/wpd4/) to obtain observed values. Data processing, non-compartmental parameter summarization, model performance evaluation, and graphical visualization were conducted in R (version 4.1, RStudio).

**FIGURE 1:**
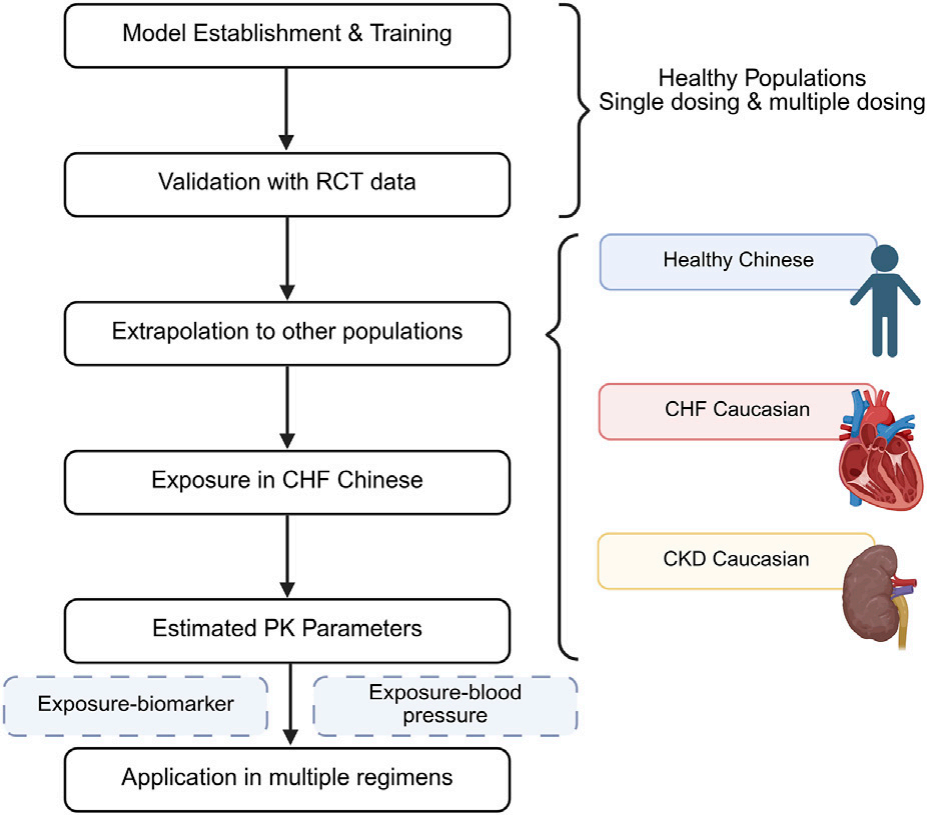
Workflow for the PBPK model of sacubitril/valsartan.

The overall workflow consisted of four sequential steps (Figure 1). First, PBPK models for sacubitril, its active metabolite sacubitrilat, and valsartan were developed and validated using published clinical pharmacokinetic data. Second, the model was extrapolated to healthy Chinese subjects and special populations, including Caucasian patients with renal impairment and CHF. Third, real-world clinical data from Chinese patients with CHF and hypertension were used to inform virtual population characteristics and clinically relevant subgroup scenarios. Finally, PBPK-predicted exposure metrics were linked to externally sourced exposure–biomarker and blood pressure response relationships to compare the expected pharmacodynamic benefit and tolerability of alternative dosing regimens.

### Clinical pharmacokinetic data

Drug-specific parameters and clinical pharmacokinetic data were obtained from literature indexed in PubMed (https://pubmed.ncbi.nlm.nih.gov), regulatory review documents from the U.S. Food and Drug Administration (https://www.fda.gov/) and DrugBank (https://www.drugbank.com). Observed plasma concentration-time profiles for sacubitril, sacubitrilat, and valsartan were extracted from published studies (Akahori et al., 2017; Gu et al., 2010; Han et al., 2017; Gan et al., 2016; Ayalasomayajula et al., 2016; Kobalava et al., 2016). Data were separated into an internal training dataset for model development and an external testing dataset for model evaluation. The training dataset included single- and multiple-dose studies spanning clinically relevant dose levels, with emphasis on 100 mg BID and the guideline-recommended 200 mg BID regimens. Details are provided in Supplementary Table S1 (Clinical studies used for PBPK model development and validation).

### Real-world clinical data

A retrospective real-world dataset of patients with hypertension and chronic heart failure treated with sacubitril/valsartan was obtained from Shanghai Minhang Hospital and Zhongshan Hospital (Ethical approval No. B2022-105). The retrospective cohort was used to inform a clinically relevant under-titrated and tolerability-limited Chinese CHF scenario rather than to estimate cohort-specific exposure–response relationships. Measured sacubitril/valsartan concentrations were not available, and longitudinal NT-proBNP, SBP, dose-titration, and discontinuation data were not collected at standardized post-dose or post-titration time points. Detailed cohort characteristics, including demographics, baseline blood pressure, renal function, LVEF, NT-proBNP, comorbidities, concomitant medications, initial sacubitril/valsartan dosing, maximum tolerated maintenance dose, and recorded adverse drug reactions, are summarized in Supplementary Table S2.

### Model structure and parameterization

Separate but linked PBPK models were constructed for sacubitril, sacubitrilat, and valsartan. After oral administration, sacubitril/valsartan was modeled as a co-administered formulation that dissociates into sacubitril and valsartan. PBPK models were built with virtual individuals generated according to demographic characteristics from each study. When demographic information was incomplete, system parameters were inferred from the PK-Sim population database.

Enzymatic conversion of sacubitril to sacubitrilat was implemented explicitly in the model. *In vivo*, sacubitril is rapidly converted to sacubitrilat by CES1 (∼76.8%). Sacubitrilat undergoes predominantly renal elimination (52%–68%), and active renal secretion has been reported to involve OAT1 and OAT3. In this model, renal active secretion was implemented as a lumped clearance process (tubular secretion) without explicit mechanistic separation of individual transporters. Valsartan undergoes minimal metabolism (∼9% via CYP2C9) and is primarily excreted unchanged. Hepatobiliary transport mediated by OATP1B1 and OATP1B3 (Hanna et al., 2018) represents the major route of its clearance. Initial parameters were optimized using a Monte Carlo approach, with details summarized in Table 1.

**TABLE 1:** Input parameters for developing sacubitril/valsartan PBPK model.

Parameter	Value	Unit	References
Sacubitril			
Physicochemical properties			
LogP	3.5	​	U.S. Food and Drug Administration (2015)
Molecular weight	411.5	g/mol	​
Fraction unbound	0.03	​	Ayalasomayajula et al. (2017)
Compound type	Acid	​	​
pKa	4.6	acidic	U.S. Food and Drug Administration (2015)
Solubility	9.01	mg/ml	Zhang et al. (2021)
Absorption			
Specific intestinal permeability	2.55E-04	cm/min	U.S. Food and Drug Administration (2015)
Distribution			
calculation method	Rodgers and Rowland	​	​
B/P	0.583	​	Lin et al. (2017)
Metabolism			
Metabolizing enzymes	CES1	​	Shi et al. (2016)
Enzyme concentration	1	mg/ml	​
Vmax	4.43	nmol/ml/min	Optimized
Km	767.2	μM	Shi et al. (2016)
Transport and Excretion			
GFR fraction	1	​	Default

Sacubitrilat			
Physiochemical properties			
LogP	2.0	​	Optimized
Molecular weight	383.444	​	​
Fraction unbound	0.03	​	Ayalasomayajula et al. (2017)
Compound type	Acid	​	​
pKa	4.585	​	CAS predicted
Solubility	0.00358	mg/ml	DrugBank
Absorption			
Specific intestinal permeability	7.10E-06	cm/min	U.S. Food and Drug Administration (2015)
Distribution			
Calculation method	Schmitt	​	Optimized
Transport and Excretion			
Transporter Vmax	0.41 (OATP1B1)	μmol/l/min	Hanna et al. (2018)
Transporter Km	116	μM	
Transporter Vmax	3.09 (OATP1B3)	μmol/l/min	
Transporter Km	311	μM	
Tubular secretion	0.46	l/min	Optimized
GFR fraction	1	​	​

Valsartan			
Physiochemical properties			
LogP	0.5	​	Optimized
Molecular weight	435.5188	​	​
Fraction unbound	0.04	​	Ayalasomayajula et al. (2017)
Compound type	Acid	​	​
pKa	3.91	​	CAS experimental
Solubility	9.32	mg/ml	Zhang et al. (2021)
Absorption			
Specific intestinal permeability	1.25E-06	cm/min	PK-sim
Distribution			
Calculation method	Poulin and Theil	​	U.S. Food and Drug Administration (2015)
Metabolism			
Metabolizing enzymes	CYP2C9	​	Nakashima et al. (2005)
Enzyme concentration	100	pmol/ml	
Vmax	27.20	pmol/ml/min	
Km	55.8	μM	
Transport and Excretion			
Transporter Vmax	0.67 (OATP1B1)	μmol/l/min	Hanna et al. (2018)
Transporter Km	4.35	μM	
Transporter Vmax	0.05 (OATP1B3)	μmol/l/min	
Transporter Km	12.1	μM	
Renal clearance	0.01	L/h/kg	Cebrián et al. (2026)
Biliary clearance	1.18	L/h/kg	Optimized

B/P ratio, blood/plasma ratio; CES, carboxylesterase; CYP, cytochrome P450; GFR, glomerular filtration rate; Km, Michaelis constant; LogP, lipophilicity; OATP, organic anion transporting polypeptide; pKa, acid dissociation constant; Vmax, maximum reaction velocity.

### Evaluation of the PBPK model

Model adequacy was assessed through graphical comparison and quantitative metrics. Simulated concentration-time profiles overlaid observed data, and the predictive consistency was inferred when observed PK metrics were encompassed within the model-generated 5^th^-95^th^ percentile intervals. Bias and precision were quantified using average fold error (AFE) and absolute fold error (AAFE) for maximum concentration (*C*_max_) and area under the concentration–time curve from the first to the last data point (AUC_0-t_). Model predictions were regarded as acceptable when the ratio of predicted to observed values remained within a two-fold boundary. Formulas to calculate FE, AFE, and AAFE are shown below.

$$FE=\frac{predicted}{observed}$$

$$AFE=10^{\frac{1}{n}*\sum \log\left(\left|\frac{{predicted}_{i}}{{observed}_{i}}\right|\right)}$$

$$AAFE=10^{\frac{1}{n}*\sum \left.\left|\log\right.\left(\frac{{predicted}_{i}}{{observed}_{i}}\right)\right|}$$

### Extrapolation to healthy Chinese and CKD patients

The PBPK model developed in healthy subjects was extrapolated to simulate pharmacokinetics in healthy Chinese individuals. Ethnicity-specific physiological differences, including organ size, blood flow, and clearance processes, were incorporated by adapting population parameters based on clinical trial baseline characteristics. A virtual cohort of 100 Chinese individuals was generated for simulation.

For chronic kidney disease (CKD), disease-related physiological changes known to affect drug disposition were implemented, including reduced renal function, prolonged intestinal transit time, and delayed gastric emptying. Virtual CKD populations were constructed based on clinical data, with severity stages defined as mild, moderate, and severe, respectively. As sacubitrilat is predominantly cleared via active tubular secretion rather than passive glomerular filtration, the standard CKD implementation based on eGFR alone may not fully capture disease-related changes in its renal disposition. Therefore, literature-informed scaling factors were introduced to modify tubular secretion clearance across CKD stages, enabling more mechanistic representation of transporter-mediated renal impairment (Hsueh et al., 2017).

### Extrapolation to CHF populations and real-world subgroups

The PBPK model was further extrapolated to CHF populations by incorporating disease-related physiological changes that may affect drug disposition. CHF-related changes included reduced cardiac output and altered regional blood flow to key organs, including the liver, kidney, heart, skeletal muscle, bone, skin, and adipose tissue (Supplementary Table S3). Additional changes affecting clearance-related processes were implemented when supported by clinical or literature evidence. Drug-specific parameters were not re-estimated during CHF extrapolation. A representative CHF virtual population was constructed rather than separate NYHA-specific models, because available clinical pharmacokinetic data did not support robust qualification of multiple CHF severity-specific PBPK models. The CHF model was evaluated using available clinical pharmacokinetic data from patients with CHF.

A virtual population of 100 CHF subjects was constructed, reflecting the demographic distribution observed in clinical data from Shanghai Minhang District Central Hospital. Subgroups were stratified according to pathophysiological status, including heart failure with coexisting hypertension and renal impairment, to capture population heterogeneity. The retrospective cohort was used to inform demographic characteristics, baseline renal function, baseline SBP distribution, subgroup definitions, and real-world dosing patterns for virtual population construction. The dataset was not used for exposure-response model development, pharmacodynamic model fitting, or clinical outcome modeling because longitudinal PK-PD and outcome data were incomplete and not collected at standardized time points. Dose-escalation scenarios (100 mg BID and 200 mg BID) were simulated, and drug exposure across subpopulations was characterized. Simulated concentration-time profiles were generated to compare pharmacokinetic differences between dosing regimens and clinical subgroups.

### Exposure–biomarker response and blood pressure effect modeling

PBPK-predicted steady-state exposure metrics were used as inputs for the pharmacodynamic component of the analysis. The efficacy-related pharmacodynamic analysis focused on the relationship between steady-state trough concentrations and NT-proBNP reduction. The exposure–biomarker relationships were not re-estimated from the real-world dataset. Instead, externally sourced relationships derived from regulatory clinical pharmacology evidence were implemented to translate PBPK-predicted exposure into expected NT-proBNP response (U.S. Food and Drug Administration and Center for Drug Evaluation and Research, 2023). The relationship between steady-state trough concentration and NT-proBNP reduction from the baseline was described using log-quadratic equations. The relationship between steady-state trough concentrations of sacubitrilat and valsartan and NT-proBNP reduction was described using the following equation:

$$R1=22.114\;*\;{\left(\log\left(C_{sac}\right)\right)}^{2}-142.29\;*\;\log\left(C_{sac}\right)+188.88$$

$$R2=15.179\;*\;{\left(\log\left(C_{val}\right)\right)}^{2}-77.39\;*\;\log\left(C_{val}\right)+63.054$$

where R1 was the reduction percentage of NT-proBNP calculated by PK parameters of sacubitrilat, R2 was the reduction percentage of NT-proBNP calculated by PK parameters of valsartan, *C*_sac_ was the steady-state trough of sacubitrilat, and *C*_val_ was the steady-state trough of valsartan.

The antihypertensive effect was characterized using a direct response model linking drug concentration to systolic blood pressure (SBP) reduction during treatment (Pool et al., 1999; Kario et al., 2014; Izzo et al., 2017) reported in the literature. Hypotension risk was assessed based on short-term changes in SBP following dose escalation, with evaluation focused on the slope of the concentration-effect relationship (Sink et al., 2018; Reed and Anderson, 1986). In addition, hypotension was defined based on expert consensus as either a reduction in SBP ≥30 mmHg or an absolute SBP <90 mmHg (Syncope Branch of Chinese Association of Geriatric Research Group of Parkinson’s Disease and Movement Disorder, 2024). The antihypertensive effect was described using the following equation:

$$E=\left(-22.5\;*\;C^{1.3}\right)/\left(200^{1.3}+C^{1.3}\right)$$

where *E* is the SBP reduction, *C* is the concentration of the drug in plasma.

Because baseline hemodynamic reserve may influence the clinical relevance of sacubitril/valsartan-associated blood pressure reduction, an exploratory baseline SBP-stratified tolerability analysis was performed. The real-world cohort was stratified according to baseline SBP distribution, and the SBP response model was applied to compare predicted SBP reduction and hypotension-related criteria under the 100 mg BID and 200 mg BID maintenance strategies.

### Sensitivity analysis

Parameter sensitivity analysis was performed using the sensitivity analysis tool provided with the PK‐Sim software. AUC_0-t_ and *C*_max_ were evaluated to identify the factors that impact the simulated PK profiles. A sensitivity value of +0.1 means that +1% change in the input parameter causes +1% change in the output PK parameter. Parameters with absolute sensitivity coefficients ≥0.1 were considered influential. To further evaluate model robustness and parameter uncertainty, a global sensitivity analysis (GSA) was conducted using the Morris screening method implemented in R. Key physiological and drug-specific parameters were simultaneously varied within predefined ranges. The Morris method was used to estimate the overall influence of each parameter on model-predicted AUC_0-t_ and *C*_max_ while accounting for parameter interactions and nonlinear effects. Parameters with larger μ* values were considered more influential. The standard deviation (σ) of elementary effects was used to assess potential parameter interactions and nonlinearity.

To evaluate the uncertainty associated with the externally sourced pharmacodynamic relationships, additional deterministic sensitivity analyses were performed for both the NT-proBNP and SBP response models. Key model parameters were varied individually by ±50% relative to their nominal values. For the NT-proBNP model, coefficients of the log-quadratic exposure-response equations were perturbed to assess their influence on predicted biomarker response. For the SBP model, Emax-related parameters were varied across the same range to evaluate the robustness of predicted blood pressure responses. The resulting changes in NT-proBNP reduction and SBP reduction were compared across dose groups.

## Results

### Demographics of real-world dataset

The retrospective real-world dataset included 131 baseline records of Chinese patients with CHF and hypertension treated with sacubitril/valsartan, with additional follow-up/event records available for selected patients. 84/130 were male (64.6%). Age was 71.9 ± 13.9 years, with a median of 73 years. Baseline systolic/diastolic blood pressure was 142.9 ± 24.7/78.5 ± 14.2 mmHg, with median values of 140/79 mmHg. Baseline eGFR was low, with a median of 39.3 mL/min/1.73 m^2^. By eGFR category, 47/131 (35.9%) were in the 30–59 range, 13/131 (9.9%) in the 15–29 range, and 41/131 (31.3%) below 15, indicating substantial renal impairment in this real-world cohort. Baseline NT-proBNP was available in 128 patients, with a median of 4385 pg/mL and a wide range, supporting substantial disease heterogeneity. 16/131 (12.2%) started sacubitril/valsartan at 25mg, 87/131 (66.4%) at 50 mg, 27/131 (20.6%) at 100 mg, and only 1/131 (0.8%) at 200 mg. The cohort was characterized by advanced age, substantial renal impairment, and predominant use of low-to-intermediate sacubitril/valsartan doses, reflecting a clinically relevant under-titrated population rather than a nationally representative CHF cohort.

### Development and validation of the PBPK model in healthy subjects

**FIGURE 2:**
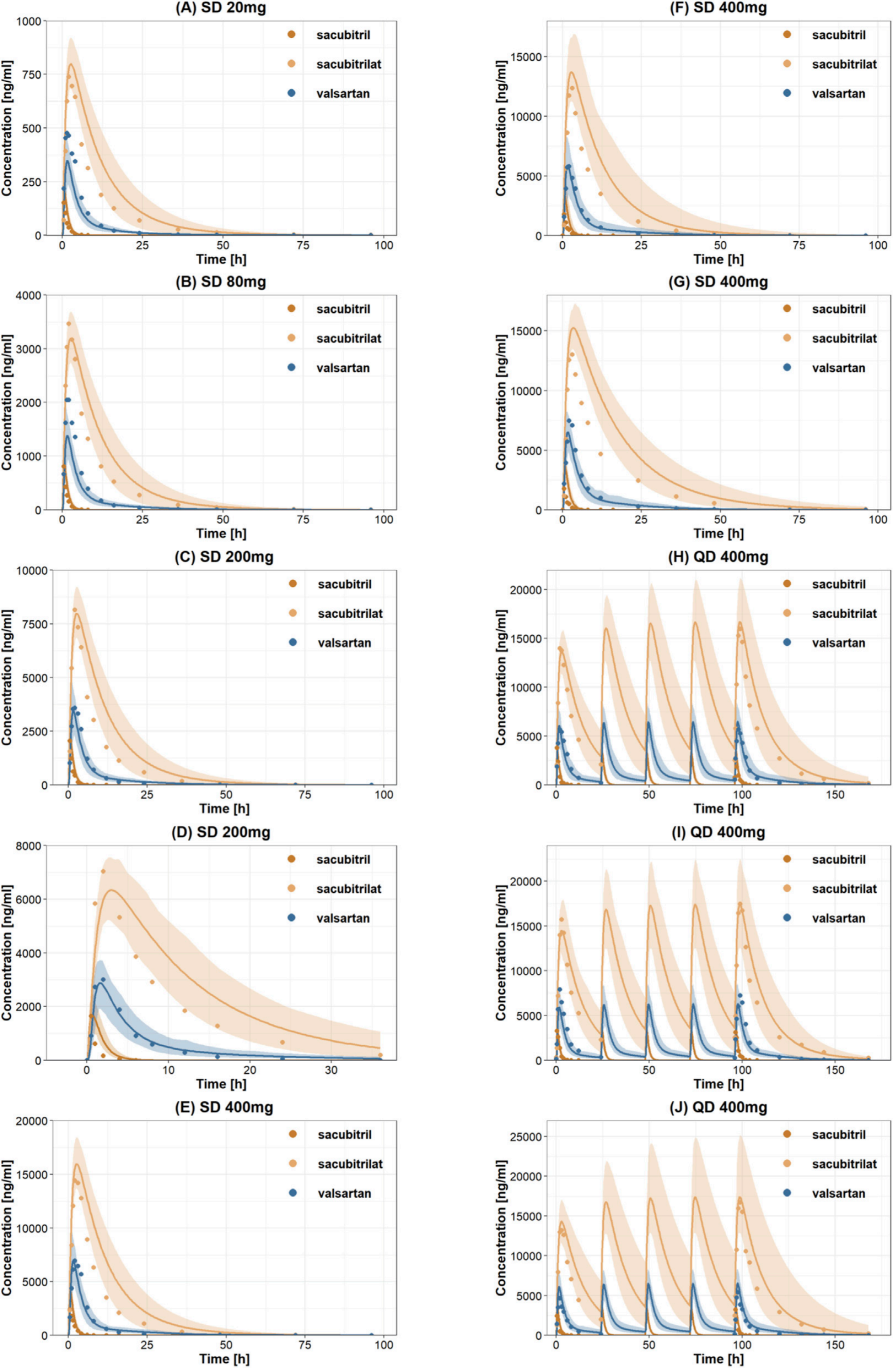
Population simulations of the PBPK model to 50, 100, 200, 400 mg oral administration. **(A–C, E, H)** training datasets. **(D, F, G, I, J)** testing datasets. Solid lines indicate the arithmetic mean of predicted plasma concentrations, dots indicate the observed data and shaded areas indicate the 5th and 95th percentiles of predicted plasma concentrations.

The PBPK model adequately described the observed plasma concentration–time profiles of sacubitril, sacubitrilat, and valsartan following both single- and multiple-dose administration. Across the training and evaluation datasets, simulated concentration–time profiles captured the observed data, with most observed concentrations falling within the simulated 5^th^–95^th^ percentile intervals (Figure 2). The model reproduced the rapid appearance and decline of sacubitril, the formation and sustained systemic exposure of sacubitrilat, and the concentration–time profile of valsartan across clinically relevant dose levels.

Model performance was further evaluated by comparing predicted-to-observed ratios of key pharmacokinetic parameters, including AUC_0-t_ and C_max_. Approximately 88.75% of the ratios were within a 1.5-fold error, and 98.75% were within a 2-fold error range (Supplementary Table S4). In addition, both AFE and AAFE were below 2. GOF plots further demonstrated close agreement between predicted and observed plasma concentrations across all single- and multiple-dose regimens (Supplementary Figure S1). Together, these results support the predictive performance of the developed PBPK model.

### Extrapolation to healthy Chinese subjects and renal impairment populations

**FIGURE 3:**
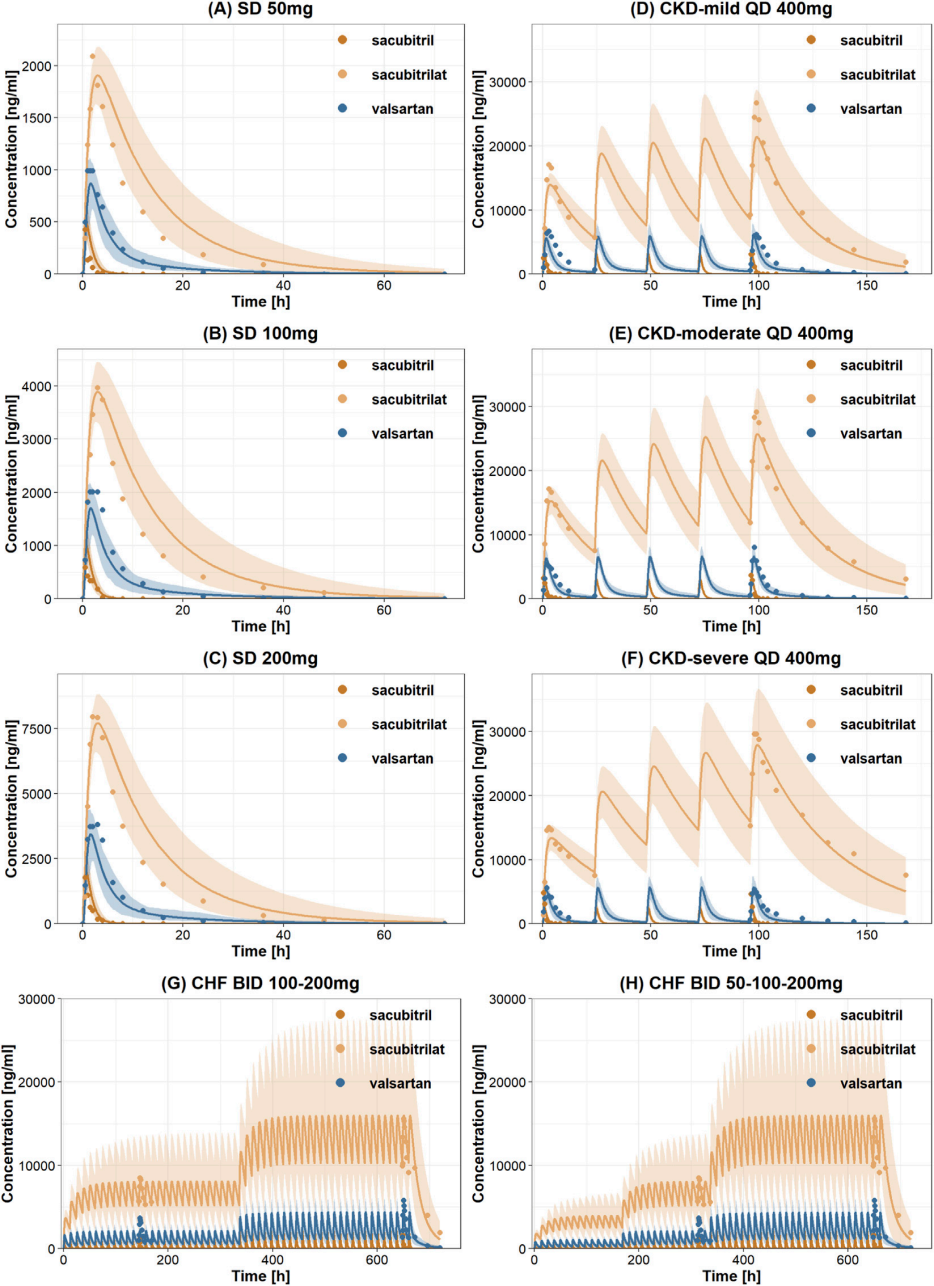
Population simulations of the PBPK model in healthy and diseased Chinese populations. **(A–C)** Healthy. **(D–F)** CKD. **(G,H)** CHF. Solid lines indicate the arithmetic mean of predicted plasma concentrations, dots indicate the observed data and shaded areas indicate the 5th and 95th percentiles of predicted plasma concentrations.

The qualified PBPK model was extrapolated to healthy Chinese subjects and patients with renal impairment. In healthy Chinese subjects, the model adequately described the observed pharmacokinetic profiles of sacubitril, sacubitrilat, and valsartan (Figures 3A–C). Compared with non-Chinese healthy populations, simulated exposure in healthy Chinese subjects was modestly higher, consistent with expected differences in body size and population physiology. However, the magnitude of difference remained within the range of interindividual variability and did not indicate a clinically meaningful exposure shift.

In renal impairment populations, the model captured the increased exposure of sacubitrilat across renal function categories (Figures 3D–F). The impact of renal impairment was most evident for sacubitrilat, consistent with its predominant renal elimination and tubular secretion component. By contrast, the exposure changes for sacubitril and valsartan were less pronounced, reflecting their different disposition pathways. Quantitative model evaluation using C_max_ and AUC showed acceptable predictive performance across healthy Chinese and renal impairment populations, with AFE and AAFE values within predefined acceptance criteria (Supplementary Table S4).

The CHF model reproduced the observed pharmacokinetic profiles of sacubitril, sacubitrilat, and valsartan in patients with heart failure (Figures 3G,H). Quantitative evaluation using predicted-to-observed ratios for C_max_ and AUC supported the predictive performance of the CHF extrapolation model (Supplementary Table S4). These results suggest that the implemented CHF-related physiological changes provided an acceptable empirical approximation for exploratory extrapolation in this population. GOF plots further demonstrated close agreement between predicted and observed plasma concentrations across all extrapolated populations (Supplementary Figure S2).

### Simulated exposure in real-world Chinese CHF subpopulations

Model predictions were further assessed in CHF subgroups, including CHF alone, CHF with hypertension (HTN), and CHF with CKD (Table 2). Across all subgroups, escalation from BID50–100 mg to BID100–200 mg resulted in an approximately dose-proportional increase in systemic exposure for sacubitril and sacubitrilat. In the CHF subgroup, sacubitrilat AUC increased from 74,065.35 ng·h/mL under the BID50–100 mg regimen to 146,623.17 ng·h/mL under the BID100–200 mg regimen, representing an approximately 98.0% increase. Sacubitrilat C_max_ similarly increased from 7,830.30 ng/mL to 15,500.74 ng/mL. Similar exposure increases were observed in the other two subgroups.

**TABLE 2:** Predicted PK parameters in real-world CHF populations.

​	PK parameters	CHF	CHF + CKD	CHF + HTN
Sacubitril				
BID50-100 mg	AUC (ng* h/ml)	2664.02	2852.32	3288.82
​	Cmax (ng/ml)	1333.3	1321.96	1481.49
BID100-200 mg	AUC (ng* h/ml)	5275.75	5647.33	6511.34
​	Cmax (ng/ml)	2640.31	2617.32	2933.1
Sacubitrilat				
BID50-100 mg	AUC (ng* h/ml)	74065.35	117538.19	76326.26
​	Cmax (ng/ml)	7830.3	11332.1	7896.02
BID100-200 mg	AUC (ng* h/ml)	146623.17	232688.47	151105.87
​	Cmax (ng/ml)	15500.74	22433.63	15630.41
Valsartan				
BID50-100 mg	AUC (ng* h/ml)	15903.0	15573.34	16110.59
​	Cmax (ng/ml)	2586.7	2622.1	2582.26
BID100-200 mg	AUC (ng* h/ml)	32077.8	31581.94	32538.56
​	Cmax (ng/ml)	5544.16	5323.03	5236.08

CKD had the most pronounced impact on sacubitrilat exposure, consistent with its predominant renal elimination pathway. Under the BID50–100 mg regimen, sacubitrilat AUC increased from 74,065.35 ng·h/mL in CHF patients to 117,538.19 ng·h/mL in CKD patients, representing an approximately 58.7% increase. Under the BID100–200 mg regimen, sacubitrilat AUC increased from 146,623.17 ng·h/mL to 232,688.47 ng·h/mL, again representing an approximately 58.7% increase. Sacubitrilat Cmax also increased substantially in CKD patients, from 7,830.30 ng/mL to 11,332.10 ng/mL under BID50–100 mg and from 15,500.74 ng/mL to 22,433.63 ng/mL under BID100–200 mg. In contrast, CKD had a less pronounced effect on sacubitril and valsartan exposure.

Hypertension did not appear to substantially affect sacubitrilat or valsartan exposure, although sacubitril exposure was numerically higher in the HTN subgroup. Overall, these simulations suggest that renal impairment, rather than hypertension alone, is the major comorbidity-related driver of increased sacubitrilat exposure in Chinese CHF patients.

### Exposure–biomarker translation for NT-proBNP response

**FIGURE 4:**
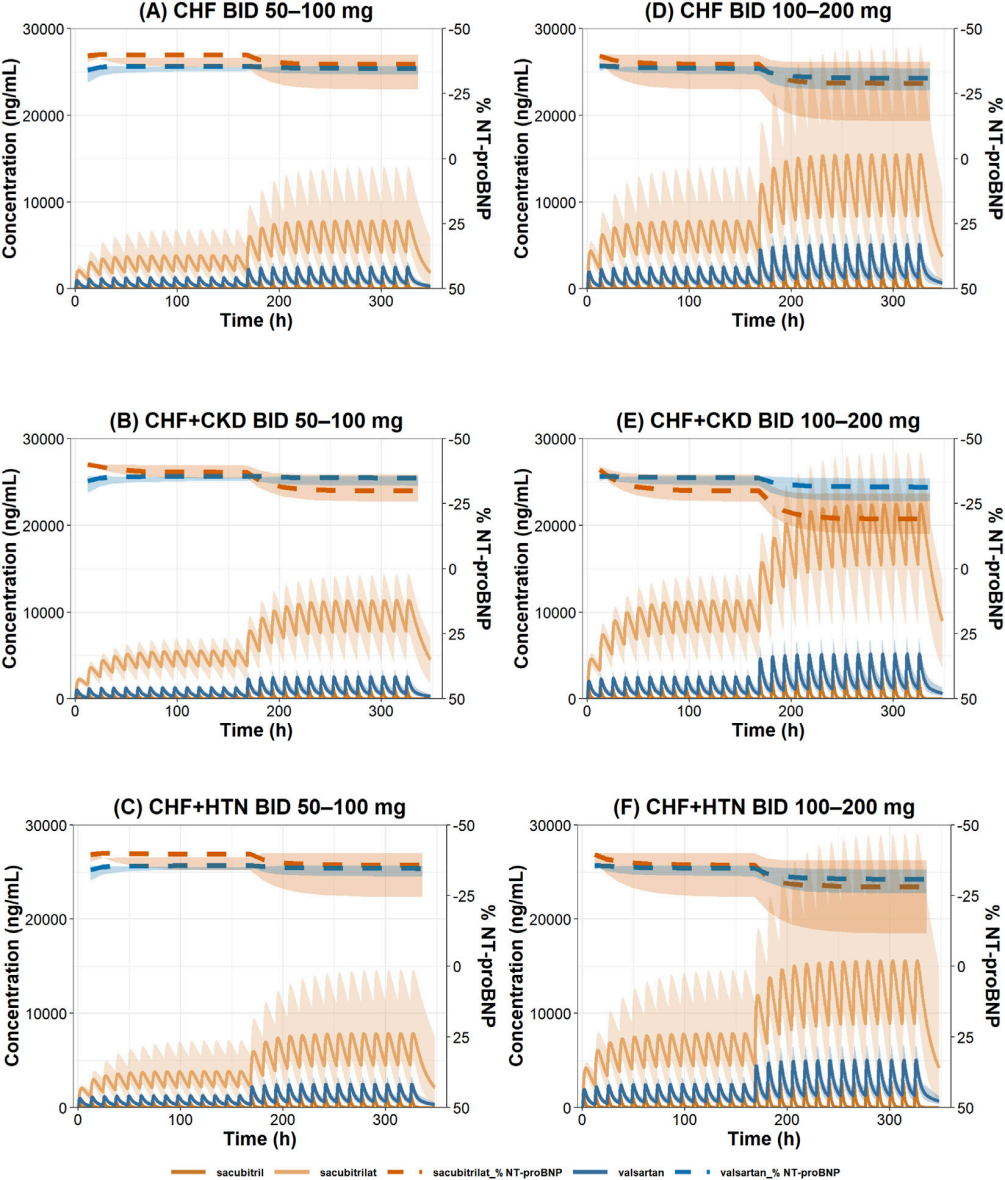
Predicted NT-proBNP reduction during simulated dose-escalation regimens in Chinese CHF subgroups. **(A,D)** CHF; **(B,E)** CHF with CKD; **(C,F)** CHF with HTN. Solid lines represent the arithmetic mean simulated plasma concentrations of sacubitril, sacubitrilat, and valsartan. Dashed lines represent the predicted sacubitrilat- and valsartan-induced percentage of NT-proBNP reduction. Shaded regions represent the 5th-95th percentile prediction intervals. Drug concentrations are shown on the left y-axis, whereas percentage of NT-proBNP reduction is shown on the right y-axis.

As shown in Figure 4, the BID100–200 mg regimen produced substantially higher sacubitrilat and valsartan exposure than the BID50–100 mg regimen across subgroups. However, the predicted NT-proBNP response showed only limited additional improvement after escalation from 100 mg BID to 200 mg BID.

In all three subgroups, the predicted NT-proBNP reduction decreased after treatment initiation and then approached a plateau. Further dose escalation was associated with a clear increase in simulated drug concentrations, particularly for sacubitrilat, but only a modest additional shift in predicted NT-proBNP reduction. This pattern was most apparent in the CKD subgroup, where sacubitrilat exposure was markedly increased, but the predicted biomarker response remained broadly overlapping with that observed under the 100 mg BID maintenance strategy.

These findings indicate a plateauing exposure–biomarker relationship over the exposure range corresponding to 100–200 mg BID. Therefore, the predicted NT-proBNP responses under 100 mg BID and 200 mg BID should be interpreted as model-predicted biomarker comparability. For Chinese CHF patients with limited tolerance to further dose escalation, maintaining 100 mg BID may preserve much of the expected NT-proBNP biomarker benefit while avoiding the additional exposure burden associated with 200 mg BID.

### Blood pressure response during dose escalation

**FIGURE 5:**
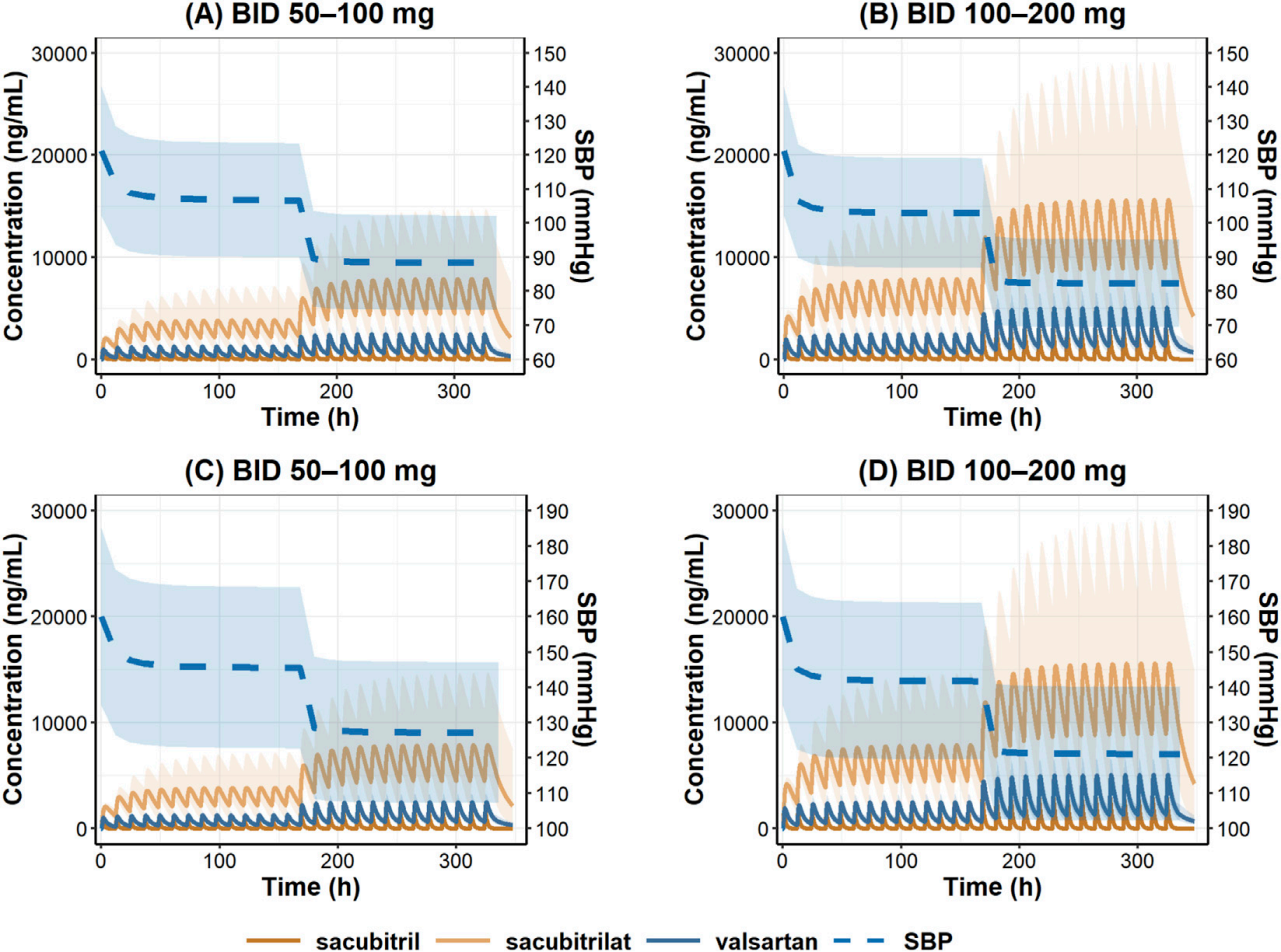
The SBP time-course profiles for regimens with dose escalation in Chinese CHF patients with HTN. Upper panel **(A,B)** indicates simulations for those with baseline SBP lower 140 mmHg, while lower panel **(C,D)** indicates above 140 mmHg baseline SBP. Solid lines represent the arithmetic mean simulated plasma concentrations of sacubitril, sacubitrilat, and valsartan. The blue dashed line represents the valsartan-induced predicted SBP response. Shaded regions represent the 5th-95th percentile prediction intervals. Drug concentrations are shown on the left y-axis, whereas SBP is shown on the right y-axis.

Model parameters demonstrated good performance in simulations informed by clinical trial data (Supplementary Figure S3). In SBP-stratified simulations, the virtual CHF population was divided into lower- and higher-baseline SBP subgroups using a threshold of 140 mmHg. The mean baseline SBP values were 121.24 mmHg and 160.04 mmHg in the lower- and higher-SBP groups, respectively. Patients with lower baseline SBP showed an increased probability of reaching hypotensive thresholds compared with the higher-SBP subgroup during dose escalation of sacubitril/valsartan (Figure 5). When considered together with the NT-proBNP exposure–biomarker analysis, these findings suggest a potential benefit–risk divergence across the evaluated dose range. Increasing the dose from 100 mg BID to 200 mg BID may produce limited additional biomarker improvement but may increase blood pressure-related tolerability risk. This suggests that 100 mg BID may be a better tolerated dosing option in Chinese CHF patients who are unable to tolerate further titration to 200 mg BID.

### Sensitivity analysis

Local sensitivity analysis was performed to evaluate the influence of model parameters on AUC and Cmax. Sacubitril exposure was predominantly affected by parameters related to its enzymatic conversion, particularly CES1-mediated metabolism, such as Km and Vmax. Consistent with its primary renal elimination pathway, sacubitrilat was most sensitive to renal-related parameters, including kidney volume and tubular secretion clearance. In contrast, valsartan exposure was mainly determined by dose and fraction unbound. Details are provided in Supplementary Figure S4. These results were consistent with the known ADME characteristics of the three analytes and supported the mechanistic plausibility of the final PBPK model.

Global sensitivity analysis (GSA) using the Morris screening method was performed to evaluate parameter influence under simultaneous variation and to capture potential nonlinear effects and interactions. For sacubitril, logP, solubility, and permeability exhibited the highest μ* and σ values, indicating that absorption-related physicochemical properties were the most influential determinants of AUC and Cmax. For sacubitrilat, fraction unbound (fu), tissue specification (TSspec), and logP showed the highest μ* values, while TSspec, fu, and logP dominated in σ. For valsartan, special clearance, dissolution, and logP demonstrated the greatest influence (Supplementary Figure S5).

Additional deterministic sensitivity analyses were performed to evaluate the robustness of the pharmacodynamic models. For the NT-proBNP response model, perturbation of the coefficients in the log-quadratic exposure-response equations affected the magnitude of the predicted biomarker reduction, with the logarithmic concentration terms showing the greatest influence. For the SBP response model, variation of Emax-related parameters altered the absolute magnitude of predicted SBP reduction. However, across the evaluated ±50% parameter ranges, the overall interpretation remained unchanged, with limited incremental NT-proBNP benefit predicted at higher doses and increased blood pressure-related tolerability concerns during dose escalation. Detailed results are provided in Supplementary Figure S6.

## Discussion

This study developed a PBPK modeling framework for sacubitril/valsartan to support dose optimization in Chinese patients with CHF. The PBPK model characterized the pharmacokinetics of sacubitril, its active metabolite sacubitrilat, and valsartan across healthy subjects, healthy Chinese subjects, renal impairment populations, and CHF patients. After model qualification using published clinical pharmacokinetic data, the model was applied to simulate exposure–biomarker and blood pressure response relationships in virtual Chinese CHF subpopulations under the assumption of transferable pharmacodynamic relationships. This framework allowed simultaneous evaluation of expected NT-proBNP reduction and blood pressure-related tolerability under alternative dose-escalation strategies.

By integrating PBPK simulations with externally sourced empirical exposure–response relationships, the model characterized both biomarker response and blood pressure-related tolerability. Predicted NT-proBNP reductions approached a plateau over the exposure range corresponding to 100–200 mg BID, suggesting limited additional short-term biomarker improvement with further dose escalation. In contrast, escalation to 200 mg BID was associated with greater predicted SBP reduction and increased hypotension-related tolerability concern. Clinical evidence indicates that dose-titration strategies influence treatment tolerability, with variation in escalation intensity and duration, and that both baseline SBP and the extent of blood pressure reduction are key determinants of hypotension risk (Senni et al., 2016; Senni et al., 2017).

The present framework may also provide a model-based perspective on the discrepancy between guideline-recommended dosing targets derived from clinical trials and real-world prescribing patterns in China. Clinical trials are designed to evaluate efficacy and safety under controlled conditions and often include dose-titration schemes with predefined tolerability criteria. In contrast, real-world CHF patients are older, more heterogeneous, and more likely to have comorbid hypertension, CKD, limited blood pressure reserve, or other factors that restrict dose escalation. As a result, many patients remain on lower maintenance doses for prolonged periods. By incorporating real-world baseline characteristics and simulating clinically relevant subgroups, this study enables exploratory assessment of individualized dose optimization in patients who are unable to reach or maintain the guideline-recommended target dose. This approach is consistent with model-informed drug development principles, which emphasize the integration of exposure–response evidence to support dose optimization and benefit–risk assessment (International Council for Harmonisation, 2026).

It is important to emphasize that the present analysis focused on short-term pharmacodynamic and tolerability markers rather than long-term clinical outcomes. NT-proBNP is a clinically meaningful biomarker of heart failure status and prognosis, and SBP is directly relevant to dose titration and treatment tolerability. However, neither endpoint can replace outcome-based evidence such as cardiovascular death, heart failure hospitalization, quality of life, treatment persistence, or long-term renal outcomes. Therefore, the predicted plateauing of NT-proBNP response between 100 mg BID and 200 mg BID should not be interpreted as evidence of long-term clinical outcome equivalence. The guideline-recommended target dose of 200 mg BID remains the preferred therapeutic target when tolerated. The present findings instead suggest that, in patients with limited tolerance to further titration, 100 mg BID may provide a feasible short-term pharmacodynamic and tolerability balance that warrants prospective validation.

Several limitations should be acknowledged. First, the NT-proBNP exposure–biomarker relationships were externally sourced from regulatory clinical pharmacology evidence rather than re-estimated using patient-level longitudinal data from the present cohort. Although additional sensitivity analyses were performed to evaluate the robustness of these pharmacodynamic relationships, the predicted magnitude of response remained dependent on empirical model assumptions, particularly those associated with the NT-proBNP log-concentration terms. Therefore, the present results should be interpreted as exploratory model-informed predictions under the assumption of transferable pharmacodynamic relationships, rather than definitive Chinese cohort-specific exposure–response evidence. Second, the PD component focused on biomarker response and blood pressure effects rather than long-term clinical outcomes, and therefore should be interpreted as hypothesis-generating rather than as evidence of comparative clinical effectiveness. Third, the representation of CHF and CKD physiology was necessarily parsimonious. CHF-related changes were implemented primarily through reduced cardiac output and altered regional organ blood flow, while CKD-related effects were represented through reduced renal function and literature-informed scaling of apparent tubular secretion clearance. Individual OAT1/OAT3 expression, uremic toxin competition, dynamic changes in eGFR, transporter regulation, CES1 variability, and NYHA severity-specific physiology were not explicitly modeled because quantitative clinical data suitable for parameterization were not available. Fourth, the retrospective cohort was not intended to represent the broader Chinese CHF population, but rather to inform virtual population construction and real-world under-titration scenarios. Consequently, the model predictions may be most applicable to tolerability-limited patients commonly encountered in routine clinical practice. Finally, the blood pressure model relied on a simplified exposure–effect relationship and predefined hypotension criteria. Circadian blood pressure variation, longitudinal treatment discontinuation, and detailed reasons for non-escalation were not modeled; future studies using prospectively collected longitudinal blood pressure, biomarker, exposure, tolerability, and clinical outcome data are needed to validate and refine this framework.

## Conclusion

In conclusion, this study established a joint modeling framework integrating sacubitril/valsartan exposure, NT-proBNP response, and blood pressure-related tolerability for exploratory evaluation of dosing strategies under-titrated in Chinese CHF patients. The findings support the guideline target of 200 mg BID when tolerated, while providing model-informed predictions suggesting that 100 mg BID may represent a potential intermediate dosing strategy for patients with tolerability limitations. This approach offers an exploratory and quantitative basis for individualized dose titration in Chinese CHF patients, particularly those with renal impairment or increased risk of hypotension.

## Data Availability

The original contributions presented in the study are included in the article/Supplementary Material, further inquiries can be directed to the corresponding authors.
