# Supplementary material for: Dose optimization of sacubitril/valsartan in Chinese chronic heart failure patients: integration of physiologically based pharmacokinetic modeling and real-world data
Source: Front Pharmacol. 2026 Jul 22;17:1875094. doi: 10.3389/fphar.2026.1875094 (PMC13437428; doi:10.3389/fphar.2026.1875094)
Supplement: Supplementary file 1 [file Supplementaryfile1.docx]

**Supplementary**

**Table S1** Clinical study used for PBPK-PD model development and validation.

| **Indicator** | **Author** | **Year** | **Population** | **Dose(mg)** | **Frequency** | **Note** |
| --- | --- | --- | --- | --- | --- | --- |
| 1 | Akahori^1^ | 2017 | Healthy Japanese | 20, 80, 200 or 400 mg | SD | Training |
| 2 | Gu^2^ | 2010 | Healthy Black | 200 mg | SD | Training |
| 3 | Han^3^ | 2017 | Healthy Chinese | 50, 100 or 200 mg | SD | Testing |
| 4 | Kobalava^4^ | 2016 | HFrEF Caucasian | 100 or 200mg | BID | Testing |
| 5 | Ayalasomayajula^5^ | 2016 | Healthy Caucasian | 400mg | QD | Training |
| 5 | Ayalasomayajula^5^ | 2016 | RI Caucasian | 400mg | QD | Testing |
| 6 | Gan^6^ | 2016 | Healthy Caucasian | 400mg | SD | Training |
| 6 | Gan^6^ | 2016 | Healthy Caucasian | 400mg | SD | Testing |

**Table S2. Baseline characteristics, comorbidities, concomitant medications, and sacubitril/valsartan dosing patterns in the retrospective real-world cohort**

| Category | Variable | Summary |
| --- | --- | --- |
| Cohort size | Baseline patient records | 131 |
|  | Unique treatment numbers | 130 |
| Demographics | Age, years | 71.9 ± 13.9; median 73.0 (66.0–82.0) |
|  | Male sex | 84/130 (64.6%) |
|  | Age <65 years | 30/130 (23.1%) |
|  | Age 65–74 years | 38/130 (29.2%) |
|  | Age ≥75 years | 62/130 (47.7%) |
|  | Age ≥80 years | 39/130 (30.0%) |
| Baseline blood pressure | SBP, mmHg | 142.9 ± 24.7; median 140.0 (125.0–160.0) |
|  | DBP, mmHg | 78.5 ± 14.2; median 79.0 (70.0–87.0) |
|  | SBP <110 mmHg | 7/131 (5.3%) |
|  | SBP <120 mmHg | 19/131 (14.5%) |
|  | SBP 120–139 mmHg | 39/131 (29.8%) |
|  | SBP ≥140 mmHg | 73/131 (55.7%) |
| Echocardiographic parameters | Left atrial diameter, mm | 45.8 ± 7.3; median 45.0 (41.0–49.0), n=113 |
|  | LVESD, mm | 36.6 ± 9.2; median 34.0 (30.3–40.0), n=118 |
|  | LVEDD, mm | 51.7 ± 7.0; median 51.0 (46.0–56.0), n=118 |
|  | Interventricular septal thickness, mm | 10.6 ± 1.8; median 10.0 (10.0–11.0), n=113 |
|  | LVEF, % | 51.8 ± 11.2; median 56.0 (44.3–61.0), n=118 |
|  | LVEF ≤40% | 24/118 (20.3%) |
|  | LVEF 41–49% | 22/118 (18.6%) |
|  | LVEF ≥50% | 72/118 (61.0%) |
| Laboratory values | ALT, U/L | 22.2 ± 23.8; median 13.0 (9.0–23.0), n=129 |
|  | AST, U/L | 31.5 ± 46.8; median 18.0 (13.0–31.0), n=129 |
|  | Serum creatinine, μmol/L | 287.1 ± 285.9; median 137.0 (92.0–410.5), n=131 |
|  | Uric acid, μmol/L | 426.5 ± 132.0; median 405.0 (335.5–516.5), n=131 |
|  | eGFR, mL/min/1.73 m² | 38.6 ± 27.4; median 39.3 (10.7–56.5), n=131 |
|  | eGFR ≥60 mL/min/1.73 m² | 30/131 (22.9%) |
|  | eGFR 30–59 mL/min/1.73 m² | 47/131 (35.9%) |
|  | eGFR 15–29 mL/min/1.73 m² | 13/131 (9.9%) |
|  | eGFR <15 mL/min/1.73 m² | 41/131 (31.3%) |
|  | Serum potassium, mmol/L | 4.0 ± 0.6; median 4.0 (3.6–4.3), n=131 |
|  | cTnT | 0.24 ± 0.44; median 0.063 (0.028–0.235), n=126 |
|  | NT-proBNP, pg/mL | 9488.9 ± 10965.9; median 4385.0 (1871.3–12556.3), n=128 |
| Comorbidities and history | Hypertension | 131/131 (100.0%) |
|  | Heart failure/cardiac insufficiency | 131/131 (100.0%) |
|  | Diabetes mellitus | 68/131 (51.9%) |
|  | Coronary artery disease | 55/130 (42.3%) |
|  | Atrial fibrillation | 32/131 (24.4%) |
|  | Renal insufficiency | 99/130 (76.2%) |
|  | Stroke/cerebral infarction history | 23/131 (17.6%) |
|  | Hyperlipidemia | 19/130 (14.6%) |
|  | Post-PCI status | 15/131 (11.5%) |
|  | Hepatic insufficiency | 8/130 (6.2%) |
|  | Smoking history | 26/131 (19.8%) |
|  | Alcohol use history | 9/131 (6.9%) |
| Concomitant medications | Diuretics | 108/131 (82.4%) |
|  | Statins | 87/130 (66.9%) |
|  | β-blockers | 76/128 (59.4%) |
|  | Antiplatelet agents | 71/131 (54.2%) |
|  | Calcium channel blockers | 60/131 (45.8%) |
|  | Antidiabetic drugs | 55/131 (42.0%) |
|  | Oral anticoagulants | 16/131 (12.2%) |
|  | α-blockers | 10/131 (7.6%) |
|  | Other cardiovascular medications | 89/131 (67.9%) |
| Initial sacubitril/valsartan dose | 25 mg | 16/131 (12.2%) |
|  | 50 mg | 87/131 (66.4%) |
|  | 100 mg | 27/131 (20.6%) |
|  | 200 mg | 1/131 (0.8%) |
|  | Initial dose ≤100 mg | 130/131 (99.2%) |
| Initial dosing frequency | BID | 119/131 (90.8%) |
|  | QD | 12/131 (9.2%) |
| Maximum tolerated maintenance dose | 25 mg | 9/130 (6.9%) |
|  | 50/51 mg | 71/130 (54.6%) |
|  | 100 mg | 47/130 (36.2%) |
|  | 200 mg | 3/130 (2.3%) |
|  | Maximum tolerated dose ≤100 mg | 127/130 (97.7%) |
| Recorded adverse drug reactions | Dizziness/vertigo | 1/131 (0.8%) |
|  | Hypotension | 4/131 (3.1%) |
|  | Other ADR | 0/131 (0.0%) |
|  | Severe ADR-related discontinuation | 0/129 (0.0%) |

**Notes:** Continuous variables are presented as mean ± SD; median (IQR). Categorical variables are presented as n/N (%). Rows with non-missing baseline serial number were treated as baseline patient records. One implausible age/sex entry was excluded from age and sex summaries. Values recorded as “>35000” for NT-proBNP were summarized as 35000 pg/mL. One maximum tolerated maintenance dose recorded as 51 mg was grouped with the 50 mg dose category for descriptive purposes.

**Table S3** Physiological changes associated with chronic heart failure.

| Parameter | Unit | Healthy | CHF | Reference |
| --- | --- | --- | --- | --- |
| Blood flow rates scale | | | | |
| Cardiac | ml/min/100g organ | 65.93 | 47.87 | Leithe1984^7^ |
| Liver | ml/min/100g organ | 17.93 | 11.50 | Leithe1984^7^ |
| Kidney | ml/min/100g organ | 193.45 | 132.51 | Leithe1984^7^ |
| Skeletal muscle | ml/min/100g organ | 3.42 | 2.13 | Leithe1984^7^ |
| Bone | ml/min/100g organ | 2.75 | 1.71 | Leithe1984^7^ |
| Skin | ml/min/100g organ | 8.65 | 5.37 | Leithe1984^7^ |
| Adipose | ml/min/100g organ | 2.18 | 1.36 | Leithe1984^7^ |
| Organ volume | | | | |
| Liver | l | 2.05 | 1.318 | Yang2024^8^ |
| Biochemical process | | | | |
| Tubular secretion | l/min | 0.46 | 0.36 | Jung2021^9^ |

**Table S4** FE, AFE and AAFE of the predicted and observed PK parameters

| Group | Parameter | Predicted | Observed | FE |
| --- | --- | --- | --- | --- |
| Sacubitril | | | | |
| SD20mg | AUC (μmol*min/l) | 49.26 | 25.81 | 1.90856 |
|  | Cmax (μmol/l) | 0.49 | 0.37 | 1.32432 |
| SD80mg | AUC (μmol*min/l) | 197.05 | 114.69 | 1.71811 |
|  | Cmax (μmol/l) | 1.97 | 1.97 | 1 |
| SD200mg | AUC (μmol*min/l) | 492.71 | 299.3 | 1.64621 |
|  | Cmax (μmol/l) | 4.92 | 5.01 | 0.98204 |
| SD400mg | AUC (μmol*min/l) | 985.66 | 543.07 | 1.81498 |
|  | Cmax (μmol/l) | 9.85 | 5.84 | 1.68664 |
| SD200mg | AUC (μmol*min/l) | 476.87 | 244.41 | 1.95111 |
|  | Cmax (μmol/l) | 4.4 | 4 | 1.1 |
| SD400mg | AUC (μmol*min/l) | 825.83 | NA |  |
|  | Cmax (μmol/l) | 8.06 | NA |  |
| SD400mg | AUC (μmol*min/l) | 1083.84 | NA |  |
|  | Cmax (μmol/l) | 9.46 | NA |  |
| QD400mg | AUC (μmol*min/l) | NA | 650.42 |  |
|  | Cmax (μmol/l) | 8.04 | 9.23 | 0.87107 |
| QD400mg | AUC (μmol*min/l) | NA | 725.6 |  |
|  | Cmax (μmol/l) | 8.61 | 8.03 | 1.07223 |
| QD400mg | AUC (μmol*min/l) | NA | 486.11 |  |
|  | Cmax (μmol/l) | 8.12 | 7.95 | 1.02138 |
| SD50mg | AUC (μmol*min/l) | 140.51 | 62.88 | 2.23457 |
|  | Cmax (μmol/l) | 1.26 | 1.03 | 1.2233 |
| SD100mg | AUC (μmol*min/l) | 286.89 | 168.41 | 1.70352 |
|  | Cmax (μmol/l) | 2.58 | 1.43 | 1.8042 |
| SD200mg | AUC (μmol*min/l) | 567.99 | 371 | 1.53097 |
|  | Cmax (μmol/l) | 5.1 | 4.31 | 1.18329 |
| CKDmild_QD400mg | AUC (μmol*min/l) | NA | 709.92 |  |
|  | Cmax (μmol/l) | 8.35 | 7.68 | 1.08724 |
| CKDmoderate_QD400mg | AUC (μmol*min/l) | NA | 635.93 |  |
|  | Cmax (μmol/l) | 9.42 | 8.95 | 1.05251 |
| CKDsevere_QD400mg | AUC (μmol*min/l) | NA | 594.07 |  |
|  | Cmax (μmol/l) | 7.93 | 11.32 | 0.70053 |
| CHF_BID100-200mg | AUC (μmol*min/l) | NA | 190.82 |  |
|  | Cmax (μmol/l) | 4.59 | 5.31 | 0.86441 |
| CHF_BID50-100-200mg | AUC (μmol*min/l) | NA | 190.82 |  |
|  | Cmax (μmol/l) | 4.59 | 5.31 | 0.86441 |
| AFE |  |  |  | 1.28 |
| AAFE |  |  |  | 1.37 |
| Sacubitrilat | | | | |
| SD20mg | AUC (μmol*min/l) | 1402.09 | 1167.67 | 1.2 |
|  | Cmax (μmol/l) | 2.02 | 1.93 | 1 |
| SD80mg | AUC (μmol*min/l) | 5608.35 | 5006.49 | 1.1 |
|  | Cmax (μmol/l) | 8.07 | 9.04 | 0.9 |
| SD200mg | AUC (μmol*min/l) | 14020.87 | 11135.8 | 1.3 |
|  | Cmax (μmol/l) | 20.18 | 21.26 | 0.9 |
| SD400mg | AUC (μmol*min/l) | 28041.65 | 21501.47 | 1.3 |
|  | Cmax (μmol/l) | 40.37 | 37.61 | 1.1 |
| SD200mg | AUC (μmol*min/l) | 13955.07 | 10722.64 | 1.3 |
|  | Cmax (μmol/l) | 17.39 | 18.36 | 0.9 |
| SD400mg | AUC (μmol*min/l) | 27460.63 | 19945.49 | 1.4 |
|  | Cmax (μmol/l) | 34.76 | 32.23 | 1.1 |
| SD400mg | AUC (μmol*min/l) | 45946.73 | 29082 | 1.6 |
|  | Cmax (μmol/l) | 40.38 | 33.92 | 1.2 |
| QD400mg | AUC (μmol*min/l) | NA | 34640.42 |  |
|  | Cmax (μmol/l) | 40.05 | 41.7 | 1 |
| QD400mg | AUC (μmol*min/l) | NA | 39525.29 |  |
|  | Cmax (μmol/l) | 43.48 | 45.53 | 1 |
| QD400mg | AUC (μmol*min/l) | NA | 36987.65 |  |
|  | Cmax (μmol/l) | 40.9 | 43.57 | 0.9 |
| SD50mg | AUC (μmol*min/l) | 4306.5 | 3110.06 | 1.4 |
|  | Cmax (μmol/l) | 4.85 | 5.45 | 0.9 |
| SD100mg | AUC (μmol*min/l) | 8792.43 | 6878.8 | 1.3 |
|  | Cmax (μmol/l) | 9.9 | 10.36 | 1 |
| SD200mg | AUC (μmol*min/l) | 17405.37 | 13514.6 | 1.3 |
|  | Cmax (μmol/l) | 19.59 | 20.73 | 0.9 |
| CKDmild_QD400mg | AUC (μmol*min/l) | NA | 102336.5 |  |
|  | Cmax (μmol/l) | 54.97 | 69.97 | 0.8 |
| CKDmoderate_QD400mg | AUC (μmol*min/l) | NA | 136521.2 |  |
|  | Cmax (μmol/l) | 70.37 | 76.08 | 0.9 |
| CKDsevere_QD400mg | AUC (μmol*min/l) | NA | 244033.2 |  |
|  | Cmax (μmol/l) | 85.7 | 77.2 | 1.1 |
| CHF_BID100-200mg | AUC (μmol*min/l) | NA | 87483.63 |  |
|  | Cmax (μmol/l) | 36.34 | 40.73 | 0.9 |
| CHF_BID50-100-200mg | AUC (μmol*min/l) | NA | 87483.63 |  |
|  | Cmax (μmol/l) | 36.34 | 40.73 | 0.9 |
| AFE |  |  |  | 1.07 |
| AAFE |  |  |  | 1.16 |
| Valsartan | | | | |
| SD20mg | AUC (μmol*min/l) | 273.34 | 405.98 | 0.7 |
|  | Cmax (μmol/l) | 1.09 | 0.87 | 1.3 |
| SD80mg | AUC (μmol*min/l) | 1096.65 | 1624.41 | 0.7 |
|  | Cmax (μmol/l) | 4.71 | 3.47 | 1.4 |
| SD200mg | AUC (μmol*min/l) | 2753.85 | 3002.43 | 0.9 |
|  | Cmax (μmol/l) | 8.68 | 8.26 | 1.1 |
| SD400mg | AUC (μmol*min/l) | 5549.73 | 5814.79 | 1 |
|  | Cmax (μmol/l) | 17.43 | 15.99 | 1.1 |
| SD200mg | AUC (μmol*min/l) | 2598.27 | 2348.53 | 1.1 |
|  | Cmax (μmol/l) | 7.55 | 6.91 | 1.1 |
| SD400mg | AUC (μmol*min/l) | 5104.05 | 5202.75 | 1 |
|  | Cmax (μmol/l) | 14.81 | 13.35 | 1.1 |
| SD400mg | AUC (μmol*min/l) | 6564.62 | 7182.37 | 0.9 |
|  | Cmax (μmol/l) | 16.6 | 17.13 | 1 |
| QD400mg | AUC (μmol*min/l) | NA | 6116.87 |  |
|  | Cmax (μmol/l) | 14.96 | 13.06 | 1.1 |
| QD400mg | AUC (μmol*min/l) | NA | 8252.73 |  |
|  | Cmax (μmol/l) | 16.26 | 16.67 | 1 |
| QD400mg | AUC (μmol*min/l) | NA | 4831.77 |  |
|  | Cmax (μmol/l) | 15.13 | 12.5 | 1.2 |
| SD50mg | AUC (μmol*min/l) | 795.81 | 781.14 | 1 |
|  | Cmax (μmol/l) | 2.18 | 2.27 | 1 |
| SD100mg | AUC (μmol*min/l) | 1535.18 | 1998.25 | 0.8 |
|  | Cmax (μmol/l) | 4.27 | 4.62 | 0.9 |
| SD200mg | AUC (μmol*min/l) | 3110.79 | 3735.81 | 0.8 |
|  | Cmax (μmol/l) | 8.62 | 8.77 | 1 |
| CKDmild_QD400mg | AUC (μmol*min/l) | NA | 10850.56 |  |
|  | Cmax (μmol/l) | 14.88 | 14.19 | 1 |
| CKDmoderate_QD400mg | AUC (μmol*min/l) | NA | 9274.88 |  |
|  | Cmax (μmol/l) | 16.98 | 18.45 | 0.9 |
| CKDsevere_QD400mg | AUC (μmol*min/l) | NA | 10588.71 |  |
|  | Cmax (μmol/l) | 15.35 | 12.27 | 1.3 |
| CHF_BID100-200mg | AUC (μmol*min/l) | NA | 11826.15 |  |
|  | Cmax (μmol/l) | 9.3 | 13.32 | 0.7 |
| CHF_BID50-100-200mg | AUC (μmol*min/l) | NA | 11826.15 |  |
|  | Cmax (μmol/l) | 9.23 | 8.49 | 1.1 |
| AFE |  |  |  | 0.98 |
| AAFE |  |  |  | 1.14 |

**Figure S1** GOF plots for sacubitril/valsartan under single-dose (SD) and multiple-dose (MD) conditions. Each point represents an AUC or Cmax ratio obtained from an individual clinical study comparison. The shaded area represents the 2-fold error boundary.

**Figure S2** GOF plots for sacubitril/valsartan in extrapolation to other populations. Each point represents an AUC or Cmax ratio obtained from an individual clinical study comparison. The shaded area represents the 2-fold error boundary.

**Figure S3** The SBP time-course profiles of validation. The red line represents the simulated valsartan plasma concentration-time profile. The blue line represents the model-predicted SBP response over time. Green dots represent observed SBP measurements extracted from the validation clinical trial.

**Figure S4** Local sensitivity analysis of AUC and Cmax of sacubitril, sacubitrilat and valsartan. Bars represent normalized local sensitivity coefficients for model parameters affecting AUC and Cmax. Positive values indicate direct relationships, whereas negative values indicate inverse relationships.

**Figure S5** Global sensitivity analysis of AUC and Cmax of sacubitril, sacubitrilat and valsartan. μ* values represent the overall influence of individual parameters on model outputs, whereas σ values indicate potential nonlinear effects and parameter interactions.

**Figure S6** Sensitivity analysis of PD parameters of NT-proBNP and SBP. The solid line represents the model prediction using nominal parameter values. The shaded regions (or dashed boundaries) represent the range of model predictions obtained by varying key pharmacodynamic parameters by ±50% relative to their baseline values.

**References:**

(1) Akahori, M.; Ayalasomayajula, S.; Langenickel, T.; Pal, P.; Zhou, W.; Sunkara, G. Pharmacokinetics After Single Ascending Dose, Food Effect, and Safety of Sacubitril/Valsartan (LCZ696), an Angiotensin Receptor and Neprilysin Inhibitor, in Healthy Japanese Subjects. *Eur J Drug Metab Pharmacokinet* **2017**, *42* (3), 407-416. DOI: 10.1007/s13318-016-0354-1 PubMed.

(2) Gu, J.; Noe, A.; Chandra, P.; Al-Fayoumi, S.; Ligueros-Saylan, M.; Sarangapani, R.; Maahs, S.; Ksander, G.; Rigel, D. F.; Jeng, A. Y.; et al. Pharmacokinetics and pharmacodynamics of LCZ696, a novel dual-acting angiotensin receptor-neprilysin inhibitor (ARNi). *J Clin Pharmacol* **2010**, *50* (4), 401-414. DOI: 10.1177/0091270009343932 PubMed.

(3) Han, Y.; Ayalasomayajula, S.; Pan, W.; Yang, F.; Yuan, Y.; Langenickel, T.; Hinder, M.; Kalluri, S.; Pal, P.; Sunkara, G. Pharmacokinetics, Safety and Tolerability of Sacubitril/Valsartan (LCZ696) After Single-Dose Administration in Healthy Chinese Subjects. *Eur J Drug Metab Pharmacokinet* **2017**, *42* (1), 109-116. DOI: 10.1007/s13318-016-0328-3 PubMed.

(4) Kobalava, Z.; Kotovskaya, Y.; Averkov, O.; Pavlikova, E.; Moiseev, V.; Albrecht, D.; Chandra, P.; Ayalasomayajula, S.; Prescott, M. F.; Pal, P.; et al. Pharmacodynamic and Pharmacokinetic Profiles of Sacubitril/Valsartan (LCZ696) in Patients with Heart Failure and Reduced Ejection Fraction. *Cardiovasc Ther* **2016**, *34* (4), 191-198. DOI: 10.1111/1755-5922.12183 PubMed.

(5) Ayalasomayajula, S. P.; Langenickel, T. H.; Jordaan, P.; Zhou, W.; Chandra, P.; Albrecht, D.; Pal, P.; Rajman, I.; Sunkara, G. Effect of renal function on the pharmacokinetics of LCZ696 (sacubitril/valsartan), an angiotensin receptor neprilysin inhibitor. *Eur J Clin Pharmacol* **2016**, *72* (9), 1065-1073. DOI: 10.1007/s00228-016-2072-7 PubMed.

(6) Gan, L.; Langenickel, T.; Petruck, J.; Kode, K.; Rajman, I.; Chandra, P.; Zhou, W.; Rebello, S.; Sunkara, G. Effects of age and sex on the pharmacokinetics of LCZ696, an angiotensin receptor neprilysin inhibitor. *J Clin Pharmacol* **2016**, *56* (1), 78-86. DOI: 10.1002/jcph.571 PubMed.
